# An Internet- and Mobile-Based Tailored Intervention to Enhance Maintenance of Physical Activity After Cardiac Rehabilitation: Short-Term Results of a Randomized Controlled Trial

**DOI:** 10.2196/jmir.3132

**Published:** 2014-03-11

**Authors:** Konstantinos Antypas, Silje C Wangberg

**Affiliations:** ^1^Norwegian Centre for Integrated Care and TelemedicineUniversity Hospital of North NorwayTromsøNorway; ^2^Department of Clinical MedicineFaculty of Health SciencesUniversity of TromsøTromsøNorway; ^3^Narvik University CollegeNarvikNorway; ^4^Regional Centre on Substance UseUniversity Hospital of North NorwayNarvikNorway

**Keywords:** rehabilitation, cardiovascular diseases, exercise therapy, eHealth, telemedicine, Internet, cellular phone, self-management, physical activity, persuasive communication, health behavior

## Abstract

**Background:**

An increase in physical activity for secondary prevention of cardiovascular disease and cardiac rehabilitation has multiple therapeutic benefits, including decreased mortality. Internet- and mobile-based interventions for physical activity have shown promising results in helping users increase or maintain their level of physical activity in general and specifically in secondary prevention of cardiovascular diseases and cardiac rehabilitation. One component related to the efficacy of these interventions is tailoring of the content to the individual.

**Objective:**

Our trial assessed the effect of a longitudinally tailored Internet- and mobile-based intervention for physical activity as an extension of a face-to-face cardiac rehabilitation stay. We hypothesized that users of the tailored intervention would maintain their physical activity level better than users of the nontailored version.

**Methods:**

The study population included adult participants of a cardiac rehabilitation program in Norway with home Internet access and a mobile phone. The participants were randomized in monthly clusters to a tailored or nontailored (control) intervention group. All participants had access to a website with information regarding cardiac rehabilitation, an online discussion forum, and an online activity calendar. Those using the tailored intervention received tailored content based on models of health behavior via the website and mobile fully automated text messages. The main outcome was self-reported level of physical activity, which was obtained using an online international physical activity questionnaire at baseline, at discharge, and at 1 month and 3 months after discharge from the cardiac rehabilitation program.

**Results:**

Included in the study were 69 participants. One month after discharge, the tailored intervention group (n=10) had a higher median level of overall physical activity (median 2737.5, IQR 4200.2) than the control group (n=14, median 1650.0, IQR 2443.5), but the difference was not significant (Kolmogorov-Smirnov *Z*=0.823, *P*=.38, *r*=.17). At 3 months after discharge, the tailored intervention group (n=7) had a significantly higher median level of overall physical activity (median 5613.0, IQR 2828.0) than the control group (n=12, median 1356.0, IQR 2937.0; Kolmogorov-Smirnov *Z*=1.397, *P*=.02, *r*=.33). The median adherence was 45.0 (95% CI 0.0-169.8) days for the tailored group and 111.0 (95% CI 45.1-176.9) days for the control group; however, the difference was not significant (*P*=.39). There were no statistically significant differences between the 2 groups in stage of change, self-efficacy, social support, perceived tailoring, anxiety, or depression.

**Conclusions:**

Because of the small sample size and the high attrition rate at the follow-up visits, we cannot make conclusions regarding the efficacy of our approach, but the results indicate that the tailored version of the intervention may have contributed to the long-term higher physical activity maintained after cardiac rehabilitation by participants receiving the tailored intervention compared with those receiving the nontailored intervention.

**Trial Registration:**

ClinicalTrials.gov: NCT01223170; http://clinicaltrials.gov/show/NCT01223170 (Archived by WebCite at http://www.webcitation.org/6Nch4ldcL).

##  Introduction

The burden of disease because of cardiovascular diseases (CVDs) has increased over the past several decades, currently ranking as the most common cause of death in Western Europe [1]. There is solid evidence that secondary prevention and cardiac rehabilitation programs can decrease the mortality risk and increase health among patients with CVD [2,3], and an important element of such interventions is engagement in physical activity [4,5]. There are different models for the delivery of secondary prevention and cardiac rehabilitation interventions, but Internet- and mobile-based platforms are very promising [6].

Internet- and mobile-based health interventions are easily accessible to many people and have the potential to influence the physical activity level of those people [7-9]. Reviews in the literature have indicated that under certain conditions such interventions can be useful tools in supporting self-management [7,10-15] and health behavior [16,17]. The effectiveness of these health interventions depends on the adoption of the appropriate theoretical framework [7,18-21], whereas the viability of these interventions is associated with strong user involvement in their design [22]. In addition, many successful interventions have utilized tailored content [9,16,22]. A tailored intervention is an intervention that is adapted to the characteristics of an individual, typically based on an individual’s responses to a questionnaire [23]. Tailored health information is generally perceived as more interesting and personally relevant, better liked, more thoroughly read and discussed, and better remembered than nontailored educational material [16,20,24-27].

We can roughly separate the technology-based cardiac rehabilitation interventions for physical activity into 2 categories. The first category aims to replace the traditional cardiac rehabilitation programs and increase the physical activity of the participants in comparison with the baseline physical activity. The second category is complementary to the traditional cardiac rehabilitation program and aims to help the users maintain their baseline level of physical activity for a longer period of time. In 2 studies that have tested the effects of such interventions by using telephone follow-up, the results have been inconsistent [28,29].

The recommended physical activity for patients in cardiac rehabilitation varies according to their risk profile, their exercise capacity, and whether the exercise training is supervised or not [2]. The general recommendation is a minimum of 2.5 hours per week of moderate aerobic activity, in multiple bouts lasting more than 10 minutes, and evenly spread throughout the week. This should be combined with the suggestion for submaximal endurance training and weight/resistance training twice a week [4]. There is evidence that aerobic interval training in short high-intensity bouts is beneficial for patients with CVD [30] and safe [31,32]. Home-based unsupervised high-intensity training was as effective and safe as supervised hospital-based training [32], but it had lower adherence. After leaving cardiac rehabilitation, patients are expected to maintain at least the recommended level of physical activity. In Northern Norway, patients are only followed up by their family doctor after discharge and there is no formal follow-up procedure by the rehabilitation center or other specialist care structure. An intervention that would support patients in maintaining their level of physical activity after their rehabilitation stay and would assist the contact and follow-up by the specialists from the rehabilitation center has the potential to facilitate the compliance with the current guidelines for cardiac rehabilitation.

The aim of our study was to assess the effect of a tailored Internet- and mobile-based intervention on the maintenance of physical activity levels after a cardiac rehabilitation stay. Our main hypothesis was that the users of the tailored intervention would maintain their level of physical activity better than the users of the nontailored intervention (control group). In our cluster randomized controlled trial (RCT), we compared a tailored version of the intervention to a nontailored version. The study design, described previously [33], allowed us to isolate the effect of tailoring and understand how and for whom the intervention worked in a real-world setting. We developed the intervention using a methodological approach that combined user input from a focus group and health behavioral theory that we have described in detail previously [34].

## Methods

### Design

The study used a 2-group cluster RCT design. The clusters were randomly assigned to either the control group, which was given access to a generic version of the website and an online forum, or the tailored group, which received the tailored intervention in addition to access to the generic content and the online forum. We used parallel groups cluster randomization based on a true random number online service. The investigators and outcome assessors were blinded to the group assignments; however, for quality assurance related to technical issues, they had to uncover the assignments early during the statistical analysis process. The participants were instructed by the personnel of the rehabilitation center to use a specific number (code) that would automatically allocate them to their monthly cluster and they were not informed of their assignment condition.

The data were collected from January 2012 until October 2013. The study measurements were made using questionnaires delivered online when the participants logged on to the Internet site while at the rehabilitation center (baseline), a short time after the planned discharge (1-3 days) from the rehabilitation center, 1 month after discharge, and 3 months after discharge (Multimedia Appendix 1). Both email and short message service (SMS) text message reminders were sent to the participants for 3 days each time they had to fill in the online questionnaire, but no further retention efforts were made. The first time the users visited the website after having received reminders about a questionnaire, they were redirected to the questionnaire. Any inconsistencies because of this were corrected to the closer follow-up time. Specifically, we analyzed at a later time point 1 response from baseline, 7 from discharge, and 4 from 1 month. Three responses from 3 months were excluded because they were closer to 1 year after discharge.

The main outcome measure was self-reported overall physical activity measured with the International Physical Activity Questionnaire (IPAQ) at 1 month and 3 months after discharge. The secondary outcome measures were self-efficacy, social support, anxiety, and depression. The process measures were the stage of change, perceived tailoring, use of the intervention, and user evaluation of the intervention.

### Participants

The participants included 69 Norwegians between the ages of 33 and 75 years recruited from Skibotn Rehabilitation Center. The inclusion criteria were (1) older than 18 years, (2) history of cardiovascular disease, (3) admission to Skibotn Rehabilitation Center, (4) access to the Internet after their stay at the rehabilitation center, and (5) possession of a personal mobile phone. The study protocol was approved by the Regional Ethics Committee for health region NORD (REK-NORD), and all participants signed a consent form before being included in the study. All participants received a present of symbolic value (a water bottle with the Web address of the intervention, NOK 50-60) if they filled in the questionnaire at 1 month after discharge. The present was offered as an incentive to use the intervention and participate in the study, but also as a token of appreciation for being part of the study. The majority of the participants were referred to the cardiac rehabilitation program by their general practitioner approximately 6 months after a hospitalization for CVD, usually after myocardial infarction.

### The Intervention

All the participants of the cardiac rehabilitation program were informed in a meeting about the study during their 4-week rehabilitation stay. Those who were interested met later to receive additional information, complete the consent form on paper, and receive training in the use of the intervention. During the training, the users registered and answered the baseline questionnaire online. The time of registration varied for the clusters. Then, the participants completed the normal rehabilitation stay, receiving no differential treatment while at the rehabilitation center. There were computers at the rehabilitation center where the participants could start using the intervention before their discharge. However, the usage was not prompted by the intervention, and the tailored component of the intervention for the tailored group was activated after discharge. Detailed description of the intervention, the tailoring algorithm, and the functionality of the intervention have been published in previous papers [33,34].

We used the free, open-source content management framework Drupal to implement all the necessary functionalities of the intervention. The intervention was provided free-of-charge to the users. The content of the website was created by the personnel of the rehabilitation center and the authors. The website was administered by one member of staff of the rehabilitation center, but most of the functionality, including the tailoring, was fully automated. We had minor changes and bug fixes to the intervention and some of the website content was updated, but because both groups used the same website, the changes affected both groups in the same way.

### Control Group

**Figure 1:**
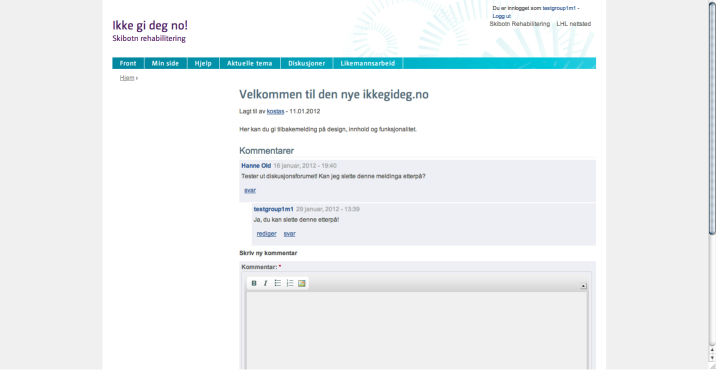
Screenshot of the discussion forum.

**Figure 2:**
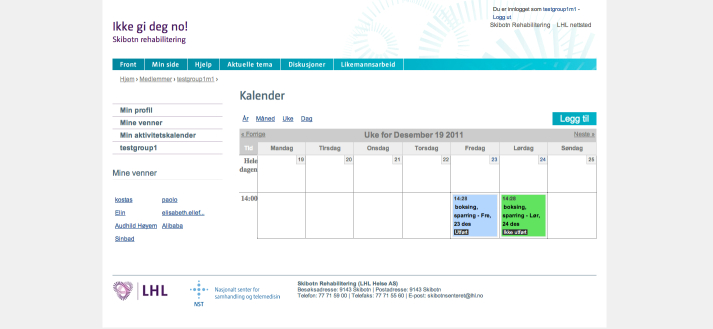
Weekly overview of the planned activities in the activity calendar. The usernames in the screenshot are of test users and not of real cases.

All the participants were given access to the basic Internet-based intervention “ikkegideg.no” (Norwegian for “Don’t give up”), which contained general information about CVD and self-management, including information about diet, physical activity, smoking, and medication, as well as access to an online discussion forum (Figure 1). In the discussion forum, there were 2 levels of access. The closed group level allowed the users to create and take part in discussions that could only be accessed by those who were members of the same monthly group. In the second, open level of access, all the users were able to create, read, and take part in discussions that were visible by all the registered users of the website. The participants of the control group were also able to plan training activities (Figure 2), but they were not prompted to do it and they received no feedback.

### Tailored Group

**Figure 3:**
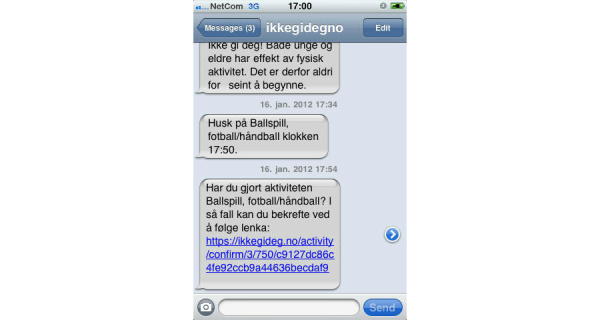
Sample SMS text message translations. Motivational (top): Don’t give up! Both young and old benefit from physical activity. Therefore, it is never too late to start. Before planned activity (middle): Remember ball game, football/handball at 17:50. After planned activity (bottom): Did you do the activity ball game, football/handball? If so, you can confirm it by following the link.

**Figure 4:**
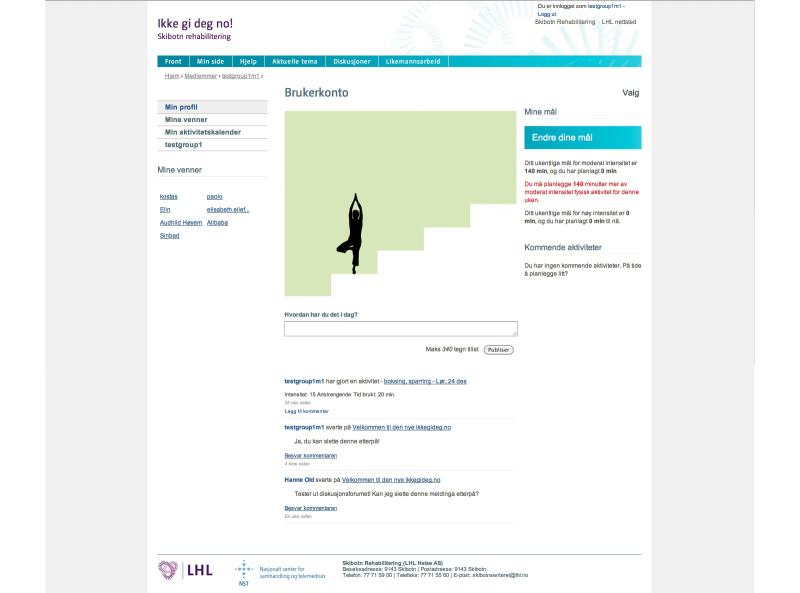
Screenshot of profile page (My Page) with a graph representing the user’s level of achievement of their weekly physical activity goals. The usernames in the screenshot are of test users and not of real cases.

The participants of the tailored group had access to the same functionality as the control group as well as access to tailored content. The participants in the tailored group were required to answer more online questions than the control group, usually every 2 weeks, and they were reminded to log in through email and SMS text messages and answer the questionnaires. Based on the tailoring questionnaires, they received tailored messages via the website and SMS text messages (Figure 3). Depending on their stage of change, the participants were asked to plan training activities or set weekly goals. They then received feedback in the form of a simple graph on the website regarding the achievement of their goals (Figure 4). If the participants planned an activity, they received an SMS text message reminder shortly before the start of the planned activity. At the end of the planned activity, they received another SMS text message asking them to confirm that the activity was completed (Figure 3). The adaptive tailoring of this intervention was based on integrative models that combined sociocognitive determinants of health behavior with a process view, such as the Health Action Process Approach (HAPA, Multimedia Appendix 2) [35]. As we have described previously [34], we tailored to the stage of change first [36], which determined if and when the other concepts were used for further tailoring (eg, self-efficacies [35,37,38] and regulatory focus [39,40]).

### Measures

The background information collected included age, gender, highest level of education, weight, and height. Physical activity was measured using the IPAQ [41,42]. Adverse events and cardiovascular outcomes were not measured. The data on use were gathered through Web logging. Our intent was to measure the number of log-ins, time spent logged in, and what elements were used most for each participant. Because of a technical issue, the time spent logged in data that we collected were not reliable. Instead, we used the time between the first and last log-in as the duration of the website use. We suspect there may have been issues with the number of log-ins per user as well, but in this case, the problem affected only a small portion of the users for a limited period of time.

The stage of change was assessed using the URICA-E2 scale [43], which gives a more comprehensive assessment of the stage than simply time before or after initiation of an action. Cronbach alpha of the 4 items that represent each stage, varied from .66 to .84. Self-efficacy was measured using the perceived competence for regular physical exercise (PC-EX) scale [44]. Responses were reported using a scale from 0 (not at all) to 6 (to a great extent). Social support was assessed using an adaptation of the scale from Barrera et al (Cronbach alpha=.93) [45].

Anxiety and depression was assessed using the Hospital Anxiety and Depression Scale (HADS), which is widely and successfully used for the postdischarge period and demonstrates satisfying diagnostic usefulness for screening depression symptoms and measuring anxiety in CVD patients [46]. There are 7 items associated with anxiety that had Cronbach alpha=.88 and 7 items for depression with Cronbach alpha=.81. The perceived tailoring was assessed using 4 items from Dijkstra [47] (Cronbach alpha=.86).

The user evaluation was assessed based on whether they would recommend the site to a friend and whether they found each of the components useful. The participants were also asked to choose the component that they found most useful and the component they found least useful from a list of the components.

### Statistical Analyses

We calculated the a priori sample size estimation with an equivalence test for 2 proportions in a cluster randomized design to detect 15% vs 5% differences in the proportion of meeting self-management behavior goals. For a .05 alpha level and 0.80 power, the required sample size was 16 clusters with 15 participants in each [33]. This sample size would be able to detect differences of 2608.1 metabolic equivalent of task (MET) min/week in the total IPAQ continuous score, a difference according to recent recommendations can result in up to 8% higher reduction in all-cause death or hospitalizations [2]. We used a standard deviation of 6095.9 MET-min/week [48], 0.015 intracluster correlation coefficient [49], and the program PASS version 12 (NCSS, Kaysville, Utah, USA). In practice, we recruited 18 clusters, but the interest of the participants within the groups was much lower than expected, resulting in an average recruitment of 3.8 participants per cluster. Because of the small size of the clusters and the variance in their size, in the following analyses we did not take into account the clusters but analyzed the population in 2 groups (tailored and control).

We tested the normality of the distribution with the Shapiro-Wilk test because the sample size was reduced to less than 50 after baseline. We found that we could not assume a normal distribution for the majority of the variables at most time points. Therefore, we report the median and the interquartile range (IQR) for the variables in each group, and we have used nonparametric methods to compare the 2 groups. Also, because of the small sample size, for the main outcome and for other continuous variables, we used the Kolmogorov-Smirnov (K-S) *Z* test with an exact calculation of the significance to compare the intervention with the control group. As an indicator of the effect size of the Kolmogorov-Smirnov *Z* comparisons, we calculated the strength of association, *r*. For the analysis of the categorical data, we used a chi-square test with an exact calculation of the significance and present the effect of the size with the phi coefficient (φ). To maximize the use of our data, we included all cases with valid data per time point and per variable.

For the analysis of adherence to the website, we used Kaplan-Meier survival curves. We used the days between the first and the last log-in and we defined “quit event” as not having used the website for the past month before the data retrieval. A Kaplan-Meier analysis can calculate the time-to-event in the presence of censored cases, such as users who are still using the website or recently recruited users. We compared the adherence curves of the tailored and control groups with the generalized Wilcoxon (Breslow) test because we expected and experienced considerably higher dropout rates at the beginning compared to the rest of the period, and the censoring patterns were similar between the groups. In contrast, when comparing the difference in adherence for gender, we used the log-rank test because we only had censored cases for the male participants.

The statistical analyses were conducted using SPSS Statistics for Mac, version 21.0 (IBM Corp, Armonk, NY, USA).

## Results

### Summary

**Figure 5:**
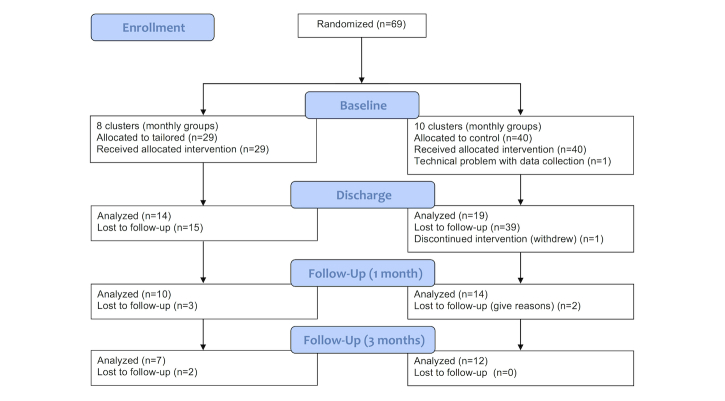
Flow diagram of the study.

The characteristics of the study participants are described in Table 1. The 2 groups were similar with respect to age, body mass index (BMI), years of education, overall physical activity (IPAQ continuous score), social support, self-efficacy, anxiety, depression, or stage of change. The flow of the participants through the study is presented in Figure 5.

**Table 1:** Baseline characteristics of the participants in the tailored and control groups.

Baseline variables		Tailored group, n=29	Control group, n=38
Age (years), mean (95% CI)		59.5 (56.3-62.8)	58.8 (55.8-61.7)
Women, n (%)		7 (24)	8 (21)
BMI, mean (95% CI)		30.4 (28.8-32.0)	29.0 (27.3-30.4)
Educational attainment (years), mean (95% CI)		13.4 (11.9-14.9)	12.4 (11.4-13.4)
Social support scale, mean (95% CI)		4.2 (3.8-4.6)	4.2 (3,8-4.6)
Self-efficacy, median (IQR)		6.0 (2.0)	5.0 (2.0)
**Baseline IPAQ scores (MET-minutes/week), median (IQR)**			
	Continuous score for walking	1386.0 (742.5)	792.00 (841.5)
	Continuous score for moderate activity	1440.0 (2400.0)	930.0 (1320.0)
	Continuous score for vigorous activity	3240.0 (4260.0)	2400.0 (2802.0)
	Continuous score for overall activity	4266.0 (6999.0)	3810.0 (3649.1)
**Hospital Anxiety and Depression Scale, median (IQR)**			
	Anxiety	4.0 (4.0)	5.0 (5.0)
	Depression	2.0 (3.5)	3.0 (4.0)
**Stage of change, n (%)**			
	Precontemplation	2 (7)	3 (8)
	Contemplation	14 (48)	17 (45)
	Preparation	1 (3)	1 (3)
	Action	6 (21)	8 (21)
	Maintenance	6 (21)	9 (24)

### Physical Activity

**Figure 6:**
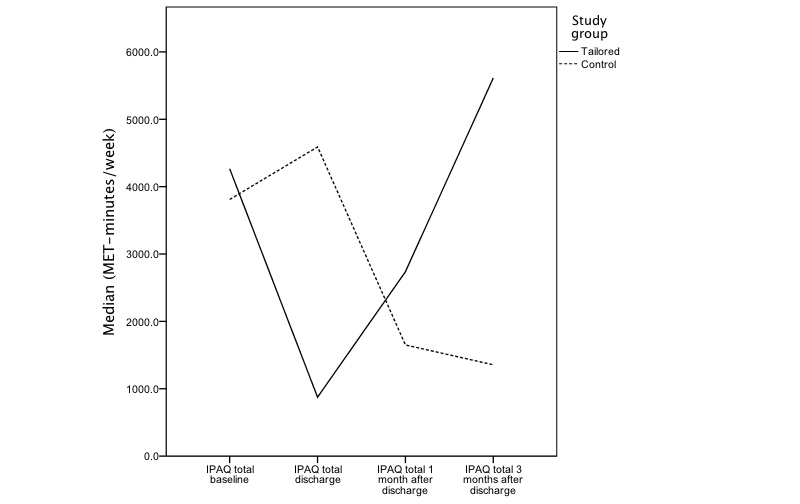
Change in International Physical Activity Questionnaire (IPAQ) total physical activity median score for each group over time.

The changes in total physical activity for each group are shown in Figure 6, and the medians and comparisons for total physical activity at each time point after baseline are shown in Table 2. One month after discharge, the overall physical activity score of the tailored group (median 2737.5, IQR 4200.2) was higher than the overall physical activity of the control group (median 1650.0, IQR 2443.5). This trend continued at 3 months after discharge with the tailored group having a significantly higher median physical activity than the control group at this time point (tailored: median 5613.0, IQR 2828.0; control: median 1356.0, IQR 2937.0).

If we look at the physical activity at different intensities, we find similar patterns Multimedia Appendix 3. Typically, the control group showed a decrease in all forms of activity at 3 months after discharge compared with the baseline value, whereas the participants in the tailored group showed an initial drop in physical activity before returning to approximately baseline levels at 3 months postdischarge (Figure 6). Three months after discharge, the tailored group had significantly higher level of walking than the control group (tailored: median 940.5, IQR 891.0; control: median 486.7, IQR 742.5), whereas the differences between the 2 groups for moderate (tailored: median 1440.0, IQR 2000.0; control: median 480.0, IQR 1080.0) and vigorous activity (tailored: median 2300.0, IQR 1824.0; control: median 0, IQR 1920.0) were not statistically significant.

For the minutes per day spent sitting, we found that at 1 month after discharge, the sitting time was higher for the control group (median 300.0, IQR 300.0) than the tailored group (median 150.0, IQR 315.0) but the difference was not significant (K-S *Z*=0.572, *P*=.61, *r*=.14). At 3 months after discharge, the tailored group showed a greater increase in sitting time than the control group, reducing the difference between the sitting times of the 2 groups (tailored: median 280.0, IQR 155.0; control: median 360.0, IQR 180.0; K-S *Z*=0.816, *P*=.43, *r*=.23).

**Table 2:** International Physical Activity Questionnaire (IPAQ) scores for total activity for the tailored and control group.

IPAQ total	Study group				Comparison test		
	Tailored		Control		K-S *Z*	*P*	*r*
	n	Median (IQR)	n	Median (IQR)			
At discharge	14	875.2 (5959.5)	19	4590.0 (3978.0)	1.473	.02	.26
At 1 month after discharge	10	2737.5 (4200.2)	13	1650.0 (2443.5)	0.823	.38	.17
At 3 months after discharge	7	5613.0 (2828.0)	11	1356.0 (2937.0)	1.397	.02	.33

### Secondary Outcomes

Self-efficacy at 1 month after discharge was the same for the tailored and the control group (tailored: median 5.0, IQR 2.0; control: median 5.0, IQR 1.0). At 3 months after discharge, the tailored group self-efficacy remained unchanged (median 5.0, IQR 2.0), but the self-efficacy of the control group increased slightly (median 5.5, IQR 2.0). The differences between the 2 groups were not statistically significant at 1 month (K-S *Z*=0.709, *P*=.27, *r*=.16) or 3 months after discharge (K-S *Z*=0.667, *P*=.36, *r*=.15).

Social support scores at 1 month after discharge, was the same for the tailored group (median 4.2, IQR 1.8) as for the control group (median 4.2, IQR 2.7). Three months after discharge, the social support of the tailored group increased (median 4.8, IQR 2.3), but it decreased in the control group (median 3.9, IQR 1.8). The difference between the groups was not significant at 1 month (K-S *Z*=0.522, *P*=.88, *r*=.12) or 3 months after discharge (K-S *Z*=0.775, *P*=.46, *r*=.19).

At 1 month after discharge, the control group experienced more anxiety than the tailored group (control: median 3.0, IQR 3.5; tailored: median 2.5, IQR 4.2). Three months after discharge, anxiety had increased for both groups, but was still higher in the control group (control: median 4.5, IQR 4.7; tailored: median 4.0, IQR 4.0). The difference in anxiety level between the groups was not statistically significant at 1 month (K-S *Z*=0.276, *P*=.98, *r*=.06) or 3 months after discharge (K-S *Z*=0.701, *P*=.44, *r*=.16).

At 1 month after discharge, depression in the control group was the same as in the tailored group (control: median 1.0, IQR 3.2; tailored: median 1.0, IQR 4.0). Three months after discharge, depression increased in both groups (control: median 1.5, IQR 2.0; tailored: median 2.0, IQR 2.0). The difference in the level of depression between the groups was not statistically significant at 1 month (K-S *Z*=0.311, *P*=.98, *r*=.06) and 3 months after discharge (K-S *Z*=0.576, *P*=.58, *r*=.13).

### Process Measures

At 1 month after discharge, 3 of 7 (43%) of the tailored group and 4 of 8 (50%) of the control group were in the action stage. Three months after discharge, 5 of 11 (45%) of the control group participants were in the action stage and 3 of 11 (27%) were in the maintenance stage, whereas 3 of 6 (50%) of the members of the tailored group were in the action stage and the other 3 (50%) were in maintenance. Overall, the participants in both groups progressed forward through the stages of change over the course of the study (Multimedia Appendix 4). There were no significant differences between the 2 groups at 1 month (χ^2^_4_=2.1, *P*=.99, φ=0.37) or 3 months after discharge (χ^2^_3_=2.2, *P*=.77, φ=0.36).

Perceived tailoring measured at 1 month after discharge was the same in the tailored (n=6, median 3.2, IQR 1.4) and the control group (n=8, median 3.2, IQR 1.6). At 3 months after discharge, the level of perceived tailoring had increased in the tailored group (n=6, median 3.6, IQR 1.4) and remained the same for the control group (n=11, median 3.2, IQR 1.7). We did not find the difference between the 2 groups statistically significant at 1 month (K-S *Z*=0.694, *P*=.60, *r*=.19) or 3 months after discharge (K-S *Z*=0.716, *P*=.39, *r*=.17).

**Figure 7:**
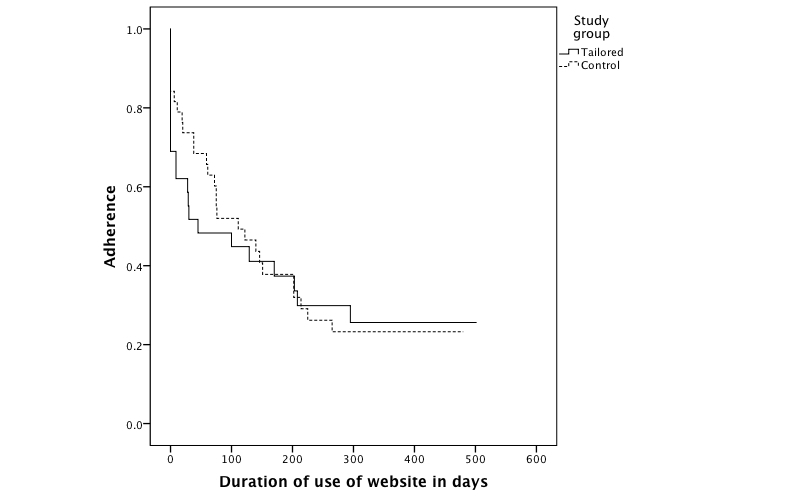
Adherence to the website.

The adherence curve was L-shaped reaching a stable use plateau at approximately 30% (Figure 7). At 1 year from baseline, the adherence rate was 25.6% for the tailored group and 24.0% for the controls. The median for adherence time for the tailored group was 45.0 (95% CI 0.0-169.8) days and 111.0 (95% CI 45.1-176.9) days for the control group; these findings were not significantly different (Breslow χ^2^_1_=0.7, *P*=.39). The median adherence time for men was 122.0 (95% CI 14.8-229.2) days and 75.0 (95% CI 0.0-153.3) days for women; these values were significantly different (log-rank χ^2^_1_=4.2, *P*=.04).

In terms of total page views, 1 month after discharge, the tailored group had visited the website more often (median 733.0, IQR 606.0) than the control group (median 392.5, IQR 464.0). However, the difference was not statistically significant (K-S *Z*=1.249, *P*=.06, *r*=.27). By 3 months after discharge, the tailored group had still visited the website more often than the control group (tailored: median 1312.0, IQR 1171.0; control: median 712.0, IQR 669.0), but the difference between the 2 groups was not significant (K-S *Z*=0.851, *P*=.38, *r*=.19).

The user evaluation was measured at 1 month after discharge. In the tailored group, 68% (6/9) of participants would recommend the website to a friend and 69% (9/13) of the control group would do likewise, although this difference was not significant (χ^2^_1_=0.02, *P*=.99). We also asked whether the participants found the different functionality elements useful. The percentages of the participants in each group that found the various functionalities useful are presented in Table 3. The most popular general functionality was goal setting (approved by 100%, 11/11 of the participants in both groups), followed by the activity calendar (approved by 100%, 6/6 of the tailored group and 90%, 9/10 of the control group), general information (approved by 83%, 5/6 of the tailored group and 80%, 8/10 of the control group) and the discussion forum (approved by 86%, 6/7 of the tailored and 73%, 8/11 of the control group). None of these differences between groups was statistically significant.

The tailored group considered the email and SMS text message reminders and messages to be the least useful functionality elements (selected by 29%, 2/7 of participants for each). The control group considered the SMS text message reminders and messages to be the least useful (selected by 20%, 2/10 of the control group participants); for the control group, these were only the reminders to complete the study questionnaires. The activity calendar was chosen as the most useful functionality by the highest proportion of users in both the tailored group (43%, 3/7) and the control group (70%, 7/10). For both the least and the most useful functionality of the intervention, the users were presented with the same list functionalities listed in Table 3.

**Table 3:** Usefulness of intervention elements.

Intervention elements		Study group, n (%)	
		Tailored	Control
**General information**			
	Yes	5 (83)	8 (80)
	No	1 (17)	2 (20)
**Discussion forum**			
	Yes	6 (86)	8 (73)
	No	1 (14)	3 (27)
**Activity calendar**			
	Yes	6 (100)	9 (90)
	No	0 (0)	1 (10)
**SMS text messages and reminders**			
	Yes	4 (67)	5 (50)
	No	2 (33)	5 (50)
**Email messages and reminders**			
	Yes	4 (67)	7 (70)
	No	2 (33)	3 (30)
**Challenge others**			
	Yes	5 (83)	7 (70)
	No	1 (17)	3 (30)
**Challenged by others**			
	Yes	5 (83)	7 (77.8)
	No	1 (17)	2 (22.2)
**My page**			
	Yes	5 (100)	9 (100)
	No	0 (0)	0 (0)
**Visit other profiles**			
	Yes	3 (60)	5 (56)
	No	2 (40)	4 (44)
**Group page**			
	Yes	3 (60)	5 (56)
	No	2 (40)	4 (44)
**Questionnaires**			
	Yes	4 (80)	7 (78)
	No	1 (20)	2 (22)
**My goals**			
	Yes	3 (100)	8 (100)
	No	0 (0)	0 (0)

##  Discussion

### Principal Findings

The intervention had high attrition rates. At the beginning of the intervention, there was a higher dropout rate in the tailored group than in the control group. The difference in average time until dropout for the 2 groups was not statistically significant. Overall, the remaining participants in our intervention moved forward through the stages of change following their rehabilitation stay; at discharge, approximately half of the participants were in the contemplation stage, whereas 3 months after discharge, half of the participants were in the action stage. Despite the fact that half of the participants received a version of the intervention that was tailored to their stage of change, there were no differences between the groups with respect to their stage progressions. There was, however, a clinically meaningful and statistically significant difference between the groups in how well they were able to maintain their total physical activity. After discharge, the tailored group began increasing their physical activity after an initial drop, whereas the control group’s physical activity decreased. This trend continued at 3 months after discharge; the physical activity of the tailored group continued to increase, whereas the physical activity of the control group continued to decline.

As the stage of change results suggest, this intervention might not have worked through the hypothesized mechanisms. The participants in the tailored group did not perceive their intervention as more personally relevant than the participants in the control group perceived theirs, and they did not consider the tailored messages received by email and SMS text message or the tailored questionnaires as particularly useful. Furthermore, the participants in the tailored group reported slightly lower self-efficacy than the control group did and approximately the same level of perceived social support as the control group.

The number of responders at 3 months was 19 of the 69 recruited at baseline (27%). This participation rate is low, but it is an expected rate for an eHealth [50] Internet-based [51] physical activity [52] intervention. There were no statistically significant differences between the 2 groups. Despite the nonsignificant difference, at the beginning of the intervention, the attrition was higher for the tailored group. A possible explanation is the increased workload of answering more questions that was required by the participants of the tailored group. The fact that the difference was not significant might be a positive sign because other studies have reported significantly higher attrition for the intervention group [53]. The dropout rate of both groups was higher at the beginning of the intervention, leading to an L-shaped adherence curve indicating that the intervention did not manage to address the needs of many users [50]. The lack of a “curiosity plateau” in the beginning, the period in which the users stay in a trial out of curiosity, might be explained by the timing of the recruitment and by the characteristics of the study population. Most of the participants of the study, especially during the beginning of their rehabilitation stay, might have been eager to employ as many methods as they could to change and maintain behavior, something that might have eased after discharge. Also, women who were interested in participating dropped out very early, significantly earlier than men. There is a known problem with cardiac rehabilitation interventions failing to address women’s needs [54-56].

Another reason for the users to stop using the intervention is that they might have achieved a satisfactory (for them) level of activity and, therefore, did not need the help of the intervention. A similar effect has been reported in smoking cessation, where nonresponders were more likely to quit than responders [57]. In an online weight management intervention, those doing light exercise were more likely to respond at 12 months than those doing moderate or vigorous exercise [58]. For the tailored group of our intervention, the algorithm would detect that the user was in the stage of maintenance, making the intervention less intensive, but for stage detection the user would have to answer some questions. If the user had already achieved a behavior, given the least effort principle, they might not see the point in spending time answering the questions. In addition, it has been found that frequency of interaction with the system might have negative impact on adherence [22]. For the nontailored group, the lack of tailoring could have made it less appealing. We can assume that because the intervention was starting immediately after discharge from the cardiac rehabilitation program, some users would already fall into the category of having an adequate level of physical activity.

A member of staff from the collaborating rehabilitation center was the website administrator, but there were no regular planned interactions by protocol. A Delphi-type study that tried to identify issues relevant to the development of an Internet-based cardiac rehabilitation intervention among specialists, found that one of the issues that scored high in relevance and consensus was the role of the cardiac case manager [59]. The frequency of interaction with a counselor was found to be a significant predictor of adherence in Web-based interventions [22]. Also, “push” factors related to researchers’ practices to keep participants in the study also have the potential to decrease dropout attrition [50], and this might be the reason that RCTs have been found to have higher adherence than larger real-life studies [22]. In our trial, we sent an SMS text message and an email reminder daily for 3 days for the research questionnaires, but we did not have any additional follow-up phone calls or actions after a dropout. Because most of the functionalities of the intervention were automated, they required little contribution from health personnel after registration, resembling a real-world sustainable scenario for such an intervention. In this way, the nonusage attrition rate of our study is an accurate estimate of the nonusage attrition rate the intervention would have if it was implemented as a routine service. Nevertheless, it is expected that increased intervention-related interaction with health professionals will improve adherence.

Problems related to user experiences might have been a reason for low adherence [50]. Even if we developed the intervention based on user needs, some elements of the intervention would not satisfy some of the users. An example is the feedback we received from some users that they would like to be able to stop receiving SMS text messages for a defined period of time, such as if they are on holiday or sick. This affects user acceptance negatively and might lead to higher attrition. A combination of methodological, economical, and technical reasons did not allow for these changes to happen. It can also be considered as more methodologically consistent to not change an important functionality of the intervention while the trial was running. However, we found that the participants, in general, were satisfied with the intervention.

The higher level of physical activity observed in the tailored group at 3 months can be primarily attributed to an increase in walking (MET-minutes/week). This difference may be due to several factors. The motivational messages that were sent to the users based on the tailoring algorithm promoted the implementation of small everyday life changes to increase physical activity, using the strategy that the participants expressed preference for in a formative focus group [34]. In addition, it may be easier for older individuals to increase their walking rather than moderate and vigorous activity [60], and individuals in Norway might prefer walking tours over other activities either on their own or in a group because of the open-air activity culture of Scandinavia [61].

Regarding the clinical relevance of our findings, the lowest group median for overall activity was observed in the tailored group at discharge (875.2 MET-min/week) and the second lowest was observed in the control group at 3 months after discharge (1356.0 MET-min/week). Thus, all the measured activity levels in our study were close to or greater than the recommended minimum limits of energy expenditure of 500-1000 MET-min/week [62]. The same guidelines, however, emphasize the importance of moderate and vigorous activity. Walking is typically categorized as a low-to-moderate activity [2,63], although its intensity can be perceived differently for different age groups [63]. Ideally, we would also like to see differences in moderate and vigorous activity to achieve levels that can predict improvements in cardiorespiratory fitness [2,4], but this does not mean that we cannot expect a benefit from the observed improvement.

To the best of our knowledge, this is the first report of an Internet- or mobile-based computer-tailored intervention targeting physical activity in cardiac rehabilitation patients. There are, however, many relevant studies of Internet-based physical activity interventions in other populations. A review of general Internet- and/or mobile-based interventions for physical activity has found consistent evidence that such programs are effective in increasing physical activity, and the most effective interventions provided tailored guidance and ongoing support [9]. Another review of Internet-based tailored health behavioral interventions that included 23 studies targeting physical activity also suggested that there is evidence for the overall efficacy of such interventions [16]. Mobile phone-based interventions to increase physical activity have been demonstrated to have a beneficial impact on influence physical activity behavior as well, especially if they are theoretically grounded [7,64].

### Strengths and Limitations

Our sample was small; therefore, our comparisons do not have enough power to confidently detect the effect of the intervention. Despite our efforts, the recruitment of participants was not at the desired levels, primarily because of the age of the participants at the cardiac rehabilitation program we recruited from. The mean age of the participants at the rehabilitation center was older than expected; therefore, interest was lower because they were less familiar with the technology we were using. In addition to the small sample size, our study was characterized by high attrition. Our study protocol did not include additional contact with the participants other than automated SMS text message and email reminders in the event of a dropout or nonusage, reflecting our choice to conduct a real-world trial of an automated system.

Furthermore, our control group received a nontailored version of the intervention, whereas the control group in other studies received usual care. This makes a difference between the groups even more difficult to detect, adding to the low statistical power problem. Although the design of our study might have decreased the statistical power, it helps us estimate if the tailored program is helpful and, if so, how it works and for whom. Our design was an effectiveness study design with the goal of isolating the effect of the tailoring rather than determining the effect of an intervention compared with a no-treatment control group.

Our approach, like that of many others [54-56], was not successful in addressing the needs of women; therefore, our results cannot be generalized to both genders. There were only a small number of women who were interested in the study and we had higher attrition rates among the women participants, contributing to the high attrition problem. Reasons that may contribute to the low adherence of women to rehabilitation programs include the tendency to minimize or play down the impact of their health situation to avoid burdening their social contacts, lower functional capacity after ischemic heart disease, and a lack of time due to family or social commitments [54]. Comorbidities, such as arthritis, osteoporosis, and urinary incontinence, can also make it harder for women to exercise [54]. At the focus group during the design phase of the intervention, women expressed their need for a service that would appeal to them too [34], but we did not receive enough information to determine what that meant and we assumed that the tailoring algorithm would address their individual needs. To increase the participation and adherence of women, we should have investigated more thoroughly any gender-specific barriers and needs.

The inclusion criteria of our study were very broad, allowing for the recruitment of participants within a wide age range with a variety of comorbidities. This makes it more difficult to demonstrate the effect of the intervention because it is more difficult to affect the health behavior of patients with more complicated cases or older people, and it is more difficult to isolate the effect of the intervention than in a carefully selected population. However, this makes our study a real-world trial that will help us understand if and how the intervention helps the population that needs it.

### Future Research

One of the major issues identified in the intervention is the high attrition. Our future research should focus more on studying attrition, and include different elements that can reduce it. Frequent interaction between a cardiac case manager and the participants seems to have great potential in improving adherence [22,59].

The addition of at least one focus group of users who used the intervention would be interesting and would complement the study. Such an approach would offer a qualitative insight into several of the quantitative findings, especially the problem of high attrition. The intervention should be developed further to include and address the needs of women, and because women are already underrepresented at the face-to-face rehabilitation program, a different approach should be used. In this case, a focus group should be organized for CVD patients after their discharge from hospital and without having participation in a cardiac rehabilitation program as a precondition.

An interesting direction for future research would be to study the effect of such an intervention before participation in a cardiac rehabilitation program. Specifically in the case of North Norway, there is a long interval between discharge from hospital and cardiac rehabilitation; therefore, an intervention such as this can be offered during this interval. This has the potential to increase recruitment to the cardiac rehabilitation program [65] because this seems to be more problematic than long-term maintenance of physical activity [66].

### Conclusion

Our main hypothesis was that participants who received a tailored intervention would maintain higher levels of physical activity over time compared to the control group. We also expected the tailored group to have better adherence to the intervention and to achieve better self-efficacy for maintenance of physical activity than the control group. The small sample size and the high attrition rate at follow-up visits in this study did not allow us to draw clear conclusions; however, the trends from our findings indicate that a tailored intervention holds promise for supporting the maintenance of long-term physical activity after cardiac rehabilitation.

## Multimedia Appendix 1

Timing of the trial.

## Multimedia Appendix 2

Video presenting an example of the tailoring algorithm.

## Multimedia Appendix 3

Intensity-specific IPAQ scores for the all the time points of the trial.

## Multimedia Appendix 4

Stage of change from discharge until 3 months after discharge.

## Multimedia Appendix 5

CONSORT-EHEALTH checklist V1.6.2 [67].

## Abbreviations

BMI: body mass index
CVD: cardiovascular disease
HADS: Hospital Anxiety and Depression Scale
HAPA: Health Action Process Approach
IPAQ: International Physical Activity Questionnaire
MET: metabolic equivalent of task
RCT: randomized controlled trial
SMS: short message service
