# Supplementary figures and images for: An Internet- and Mobile-Based Tailored Intervention to Enhance Maintenance of Physical Activity After Cardiac Rehabilitation: Short-Term Results of a Randomized Controlled Trial
Source: J Med Internet Res. 2014 Mar 11;16(3):e77. doi: 10.2196/jmir.3132 (PMC3967125; doi:10.2196/jmir.3132)

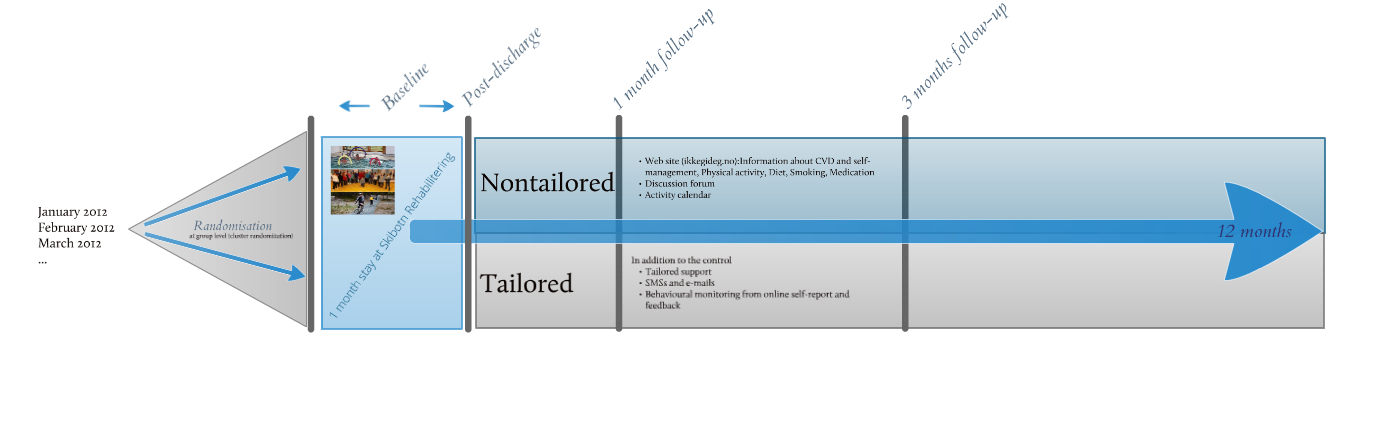

Supplement: Supplementary file 1 [file jmir_v16i3e77_app1.jpg]
