# Supplementary material for: An Internet- and Mobile-Based Tailored Intervention to Enhance Maintenance of Physical Activity After Cardiac Rehabilitation: Short-Term Results of a Randomized Controlled Trial
Source: J Med Internet Res. 2014 Mar 11;16(3):e77. doi: 10.2196/jmir.3132 (PMC3967125; doi:10.2196/jmir.3132)
Supplement: Supplementary file 3 [file jmir_v16i3e77_app3.pdf]

	Study group						
	Tailored			Control			
	n	Median	IQR	n	Median	IQR	Comparison test
IPAQ walk at discharge	12	635.2	1212.7	15	693.0	511.5	K-S Z=0.732, *p=0,494,* r=0.14
IPAQ walk at 1 month after discharge	10	594.0	726.0	12	544.5	730.1	K-S Z=0.272, *p=0.995,* r=0.06
IPAQ walk at 3 months after discharge	6	940.5	891.0	10	486.7	742.5	K-S Z=1.226, *p=0.050,* r=0.31
IPAQ moderate at discharge	12	240.0	1620.0	17	1200.0	2700.0	K-S Z=1.014, *p=0.181,* r=0.19
IPAQ moderate at 1 month after discharge	9	720.0	1070.0	12	0	1860.0	K-S Z=0.756, *p=0.431,* r=0.16
IPAQ moderate at 3 months after discharge	7	1440.0	2000.0	9	480.0	1080.0	K-S Z=0.976, *p=0.209,* r=0.24
IPAQ vigorous at discharge	12	840.0	3600.0	18	2400.0	2120.0	K-S Z=1.043, *p=0.169,* r=0.19
IPAQ vigorous at 1 month after discharge	10	1200.0	2340.0	11	480.0	1416.0	K-S Z=0.728, *p=0.446,* r=0.16
IPAQ vigorous at 3 months after discharge	6	2300.0	1824.0	11	0	1920.0	K-S Z=1.134, *p=0.075,* r=0.27
