# Supplementary material for: An Internet- and Mobile-Based Tailored Intervention to Enhance Maintenance of Physical Activity After Cardiac Rehabilitation: Short-Term Results of a Randomized Controlled Trial
Source: J Med Internet Res. 2014 Mar 11;16(3):e77. doi: 10.2196/jmir.3132 (PMC3967125; doi:10.2196/jmir.3132)
Supplement: Supplementary file 4 [file jmir_v16i3e77_app4.pdf]

	Study group				
	Tailored		Control		
	Median	IQR	Median	IQR	Comparison test
Self-efficacy at discharge	5.0	2.0	5.0	2.0	K-S Z=0.505, *p=0.521*, r=0.09
Self-efficacy at 1 month after discharge	5.0	2.0	5.0	1.0	K-S Z=0.709, *p=0.273*, r=0.16
Self-efficacy at 3 months after discharge	5.0	2.0	5.5	2.0	K-S Z=0.667, *p=0.365*, r=0.15
