# Supplementary material for: An Internet- and Mobile-Based Tailored Intervention to Enhance Maintenance of Physical Activity After Cardiac Rehabilitation: Short-Term Results of a Randomized Controlled Trial
Source: J Med Internet Res. 2014 Mar 11;16(3):e77. doi: 10.2196/jmir.3132 (PMC3967125; doi:10.2196/jmir.3132)
Supplement: Supplementary file 5 [file jmir_v16i3e77_app5.pdf]

CONSORT-EHEALTH Checklist V1.6.2 Report

(based on CONSORT-EHEALTH V1.6), available at [http://tinyurl.com/consort-ehealth-v1-6].

3132

Date completed

11/25/2013 15:02:37

by

Konstantinos Antypas

An Internet- and mobile-based tailored intervention to enhance maintenance of physical activity after cardiac rehabilitation: short-term results of a randomized controlled trial

TITLE

1a-i) Identify the mode of delivery in the title

"Internet- and mobile-based tailored intervention"

1a-ii) Non-web-based components or important co-interventions in title

"after cardiac rehabilitation"

1a-iii) Primary condition or target group in the title

"cardiac rehabilitation" This is broad but it reflects the inclusion of all type of cardiac rehabilitation patients.

ABSTRACT

1b-i) Key features/functionalities/components of the intervention and comparator in the METHODS section of the ABSTRACT

"The participants were randomized in monthly clusters to a tailored or non-tailored (control) intervention group. All of the participants had access to a website with information regarding cardiac rehabilitation, an online discussion forum and an online activity calendar. In addition, those randomized to the tailored intervention received tailored content based on models of health behavior via the website and mobile fully automated text messages."

1b-ii) Level of human involvement in the METHODS section of the ABSTRACT

"fully automated text messages"

1b-iii) Open vs. closed, web-based (self-assessment) vs. face-to-face assessments in the METHODS section of the ABSTRACT

"The study population included adult participants of a cardiac rehabilitation program in Norway"

"The main outcome was the self-reported level of physical activity measured in MET-minutes per week, which was obtained using an online international physical activity questionnaire "

"participants were blinded to the group assignments."

1b-iv) RESULTS section in abstract must contain use data

"Included in the study were 69 participants. At one month after discharge, we analyzed 10 users in the tailored intervention group and 14 in the control group, and at three months after discharge, we analyzed 7 users in the tailored and 12 in the control group. "

"The mean adherence was 176.3 (100.3–252.4) days for the tailored group and 177.9 (119.1-236.6) for the control group; however, the difference was not significant (Breslow $\chi 2=0.725$, $p=0.395$)."

1b-v) CONCLUSIONS/DISCUSSION in abstract for negative trials

N/A

INTRODUCTION

2a-i) Problem and the type of system/solution

"The aim of our study was to assess the effect of a tailored Internet- and mobile-based intervention on the maintenance of physical activity levels after a cardiac rehabilitation stay."

2a-ii) Scientific background, rationale: What is known about the (type of) system

"Internet- and mobile-based health interventions are easily accessible to many people and have the potential to influence the physical activity level of those people [7–9]. Reviews in the literature have indicated that under certain conditions, such interventions can be useful tools in supporting self-management [7,10–15] and health behavior [16,17]. The effectiveness of these health interventions depends on the adoption of the appropriate theoretical framework [7,18–21], whereas the viability of these interventions is associated with strong user involvement in their design [22]. In addition, many successful interventions have utilized tailored content [9,16,22]. A tailored intervention is an intervention that is adapted to the characteristics of an individual, typically based on an individual's responses to a questionnaire [23]. Tailored health information is generally perceived as more interesting and personally relevant, better liked, more thoroughly read and discussed, and better remembered than non-tailored educational material [16,20,24–27]."

METHODS

3a) CONSORT: Description of trial design (such as parallel, factorial) including allocation ratio

"Our main hypothesis was that the users of the tailored intervention would maintain their level of physical activity better than the users of the non-tailored intervention (control group). In our cluster randomized controlled trial, we compared a tailored version of the intervention with a non-tailored version."

3b) CONSORT: Important changes to methods after trial commencement (such as eligibility criteria), with reasons

"Because of the small size of the clusters and the variance in their size, in the following analyses we did not take into account the clusters but analyzed the population in two groups (tailored and control).2

3b-i) Bug fixes, Downtimes, Content Changes

"Our intent was to measure the number of logins, time spent logged in, and what elements were used most for each participant. Due to a technical issue, the "time spent logged-in" data that we collected was not reliable. Instead, we used the time between the first and last login as the duration of the website use. We suspect there may have been issues with the number of logins per user as well, but in this case, the problem affected only a small portion of the users for a limited period of time."

4a) CONSORT: Eligibility criteria for participants

"The inclusion criteria were (1) older than 18, (2) history of cardiovascular disease, (3) admission to Skibotn Rehabilitation Center, (4) access to the Internet after their stay at the rehabilitation center, and (5) possession of a personal mobile phone."

4a-i) Computer / Internet literacy

"4) access to the Internet after their stay at the rehabilitation center"

" Those who were interested met later to receive additional information, complete the consent form and receive training in the use of the intervention"

4a-ii) Open vs. closed, web-based vs. face-to-face assessments:

"recruited from Skibotn Rehabilitation Center"

"The study measurements were made using questionnaires delivered online "

4a-iii) Information giving during recruitment

"All the participants of the cardiac rehabilitation program were informed in a meeting about the study during their four-week rehabilitation stay. Those who were interested met later to receive additional information, complete the consent form and receive training in the use of the intervention. "

4b) CONSORT: Settings and locations where the data were collected
"The study measurements were made using questionnaires delivered online when the participants logged on to the Internet site while at the rehabilitation center (baseline), a short time after the planned discharge (1-3 days) from the rehabilitation center, one month after discharge, and three months after discharge. "
4b-i) Report if outcomes were (self-)assessed through online questionnaires
"The study measurements were made using questionnaires delivered online when the participants logged on to the Internet site while at the rehabilitation center (baseline), a short time after the planned discharge (1-3 days) from the rehabilitation center, one month after discharge, and three months after discharge. "
4b-ii) Report how institutional affiliations are displayed
We don't think this was a bias. Recruitment was from face-to-face program and the study was presented as a project of the the face-to-face program.
5) CONSORT: Describe the interventions for each group with sufficient details to allow replication, including how and when they were actually administered
5-i) Mention names, credential, affiliations of the developers, sponsors, and owners
"We used the free, open-source content management framework Drupal to implement all of the necessary functionalities of the intervention."
5-ii) Describe the history/development process
"We developed the intervention using a methodological approach that combines user input from a focus group and health behavioral theory that we have described previously in detail [32]."
5-iii) Revisions and updating
"We had minor changes and bug fixes on the intervention, and some of the website content was updated, but since both groups were using the same website, the changes affected both groups in the same way."
5-iv) Quality assurance methods
N/A
5-v) Ensure replicability by publishing the source code, and/or providing screenshots/screen-capture video, and/or providing flowcharts of the algorithms used
Screenshots are submitted and detailed description of theories used are mentioned in this paper and in other referenced paper. Free open-source platform used.
5-vi) Digital preservation
Screenshots are submitted. Free open-source platform used.
5-vii) Access
"All of the participants were given access to the basic Internet-based intervention, “ikkegideg.no” (Norwegian for “Don’t give up”), "
"The intervention was provided free-of-charge to the users."
5-viii) Mode of delivery, features/functionalities/components of the intervention and comparator, and the theoretical framework
"Control group
All of the participants were given access to the basic Internet-based intervention, “ikkegideg.no” (Norwegian for “Don’t give up”), which contained general information about CVD and self-management, including information about diet, physical activity, smoking, and medication, as well as access to an online discussion forum (Figure 1). In the discussion forum, there were two levels of access. The closed group level allowed the users to create and take part in discussions that could only be accessed by those who were members of the same monthly group. In the second, open level of access, all of the users were able to create, read and take part in discussions that were visible by all of the registered users of the website. The participants of the control group were also able to plan training activities (Figure 2) but were not prompted or reminded to do it and received no feedback.
Tailored group
The participants of the tailored group had access to the same functionality as the control group as well as access to tailored content. The participants in the tailored group were required to answer more online questions than the control group, usually every two weeks, and received tailored messages via the website and Short Message Service (SMS) (Figure 3). Depending on their stage of change, the participants were asked to plan training activities or set weekly goals. They then received feedback in the form of a simple graph on the website regarding the achievement of their goals (Figure 4). If the participants planned an activity, they received an SMS reminder shortly before the start of the planned activity. At the end of the planned activity, they received another SMS asking them to confirm that the activity was completed (Figure 3). The adaptive tailoring of this intervention was based on integrative models that combined socio-cognitive determinants of health behavior with a process view, such as the Health Action Process Approach HAPA (Multimedia Appendix 1)[33]. As we have described previously [32], we tailored first to the stage of change [34], which then determined if and when the other concepts were used for further tailoring (e.g., self-efficacies [33,35,36] and regulatory focus [37,38])."
5-ix) Describe use parameters
No recommendations about the use.
5-x) Clarify the level of human involvement
"The content of the website was created by the personnel of the rehabilitation centre and the authors. The website was administered by one member of staff of the rehabilitation centre but most of the functionality, including the tailoring, was fully automated."
5-xi) Report any prompts/reminders used
"E-mail and SMS reminders were sent to the participants for three days each time they had to fill in the online questionnaire."
5-xii) Describe any co-interventions (incl. training/support)
"Tailored group
The participants of the tailored group had access to the same functionality as the control group as well as access to tailored content. The participants in the tailored group were required to answer more online questions than the control group, usually every two weeks, and received tailored messages via the website and Short Message Service (SMS) (Figure 3). Depending on their stage of change, the participants were asked to plan training activities or set weekly goals. They then received feedback in the form of a simple graph on the website regarding the achievement of their goals (Figure 4). If the participants planned an activity, they received an SMS reminder shortly before the start of the planned activity. At the end of the planned activity, they received another SMS asking them to confirm that the activity was completed (Figure 3). The adaptive tailoring of this intervention was based on integrative models that combined socio-cognitive determinants of health behavior with a process view, such as the Health Action Process Approach HAPA (Multimedia Appendix 1)[33]. As we have described previously [32], we tailored first to the stage of change [34], which then determined if and when the other concepts were used for further tailoring (e.g., self-efficacies [33,35,36] and regulatory focus [37,38])."
6a) CONSORT: Completely defined pre-specified primary and secondary outcome measures, including how and when they were assessed

"The main outcome measure was self-reported physical activity. The secondary outcome measures were self-efficacy, social support, anxiety and depression, and the process measures were the stage of change, perceived tailoring, use of the intervention, and user evaluation of the intervention."
"The background information collected included age, gender, highest level of education, weight and height. Physical activity was measured using the International Physical Activity Questionnaire IPAQ [39,40]. The data on use were gathered through web logging. Our intent was to measure the number of logins, time spent logged in, and what elements were used most for each participant. Due to a technical issue, the "time spent logged-in" data that we collected was not reliable. Instead, we used the time between the first and last login as the duration of the website use. We suspect there may have been issues with the number of logins per user as well, but in this case, the problem affected only a small portion of the users for a limited period of time.
The stage of change was assessed using the URICA-E2 scale [41], which gives a more comprehensive assessment of the stage than simply time before or after initiation of an action. Cronbach's α, which measures the internal consistency of the four items that represent each stage, varied from 0.66 to 0.84. Self-efficacy was measured using the perceived competence for regular physical exercise (PC-EX) scale [42]. The responses were reported using a scale from 0 (not at all) to 6 (to a great extent). Social support was assessed using an adaptation of the scale from Barrera et al., which had excellent internal consistency (Cronbach's α=0.93) [43].
Anxiety and Depression was assessed using the Hospital Anxiety and Depression Scale (HADS), which is widely and successfully used for the post-discharge period and demonstrates satisfying diagnostic usefulness for screening depression symptoms and measuring anxiety in CVD patients [44]. There are seven items associated with anxiety that had good internal consistency (Cronbach's α=0.88) and seven items for depression with good internal consistency (Cronbach's α=0.81). The perceived tailoring was assessed using four items from Dijkstra that showed good internal consistency (Cronbach's α=0.86) [45].
The user evaluation was assessed based on whether they would recommend the site to a friend and whether they found each of the components useful. The participants were also asked to choose the most and least useful components. "

6a-i) Online questionnaires: describe if they were validated for online use and apply CHERRIES items to describe how the questionnaires were designed/deployed

CHERRIES was not used to describe.
"The background information collected included age, gender, highest level of education, weight and height. Physical activity was measured using the International Physical Activity Questionnaire IPAQ [39,40]. The data on use were gathered through web logging. Our intent was to measure the number of logins, time spent logged in, and what elements were used most for each participant. Due to a technical issue, the "time spent logged-in" data that we collected was not reliable. Instead, we used the time between the first and last login as the duration of the website use. We suspect there may have been issues with the number of logins per user as well, but in this case, the problem affected only a small portion of the users for a limited period of time.
The stage of change was assessed using the URICA-E2 scale [41], which gives a more comprehensive assessment of the stage than simply time before or after initiation of an action. Cronbach's α, which measures the internal consistency of the four items that represent each stage, varied from 0.66 to 0.84. Self-efficacy was measured using the perceived competence for regular physical exercise (PC-EX) scale [42]. The responses were reported using a scale from 0 (not at all) to 6 (to a great extent). Social support was assessed using an adaptation of the scale from Barrera et al., which had excellent internal consistency (Cronbach's α=0.93) [43].
Anxiety and Depression was assessed using the Hospital Anxiety and Depression Scale (HADS), which is widely and successfully used for the post-discharge period and demonstrates satisfying diagnostic usefulness for screening depression symptoms and measuring anxiety in CVD patients [44]. There are seven items associated with anxiety that had good internal consistency (Cronbach's α=0.88) and seven items for depression with good internal consistency (Cronbach's α=0.81). The perceived tailoring was assessed using four items from Dijkstra that showed good internal consistency (Cronbach's α=0.86) [45].
The user evaluation was assessed based on whether they would recommend the site to a friend and whether they found each of the components useful. The participants were also asked to choose the most and least useful components."

6a-ii) Describe whether and how "use" (including intensity of use/dosage) was defined/measured/monitored

"The data on use were gathered through web logging. Our intent was to measure the number of logins, time spent logged in, and what elements were used most for each participant. Due to a technical issue, the "time spent logged-in" data that we collected was not reliable. Instead, we used the time between the first and last login as the duration of the website use. We suspect there may have been issues with the number of logins per user as well, but in this case, the problem affected only a small portion of the users for a limited period of time."

6a-iii) Describe whether, how, and when qualitative feedback from participants was obtained

No qualitative feedback.

6b) CONSORT: Any changes to trial outcomes after the trial commenced, with reasons

N/A

7a) CONSORT: How sample size was determined

7a-i) Describe whether and how expected attrition was taken into account when calculating the sample size

"We calculated the a priori sample size estimation with an equivalence test for two proportions in a cluster-randomized design to detect 15% vs. 5% differences in the proportion of meeting self-management behavior goals. For a 0.05 alpha level and a 0.80 power, the required sample size was 16 clusters with 15 participants in each. In practice, we recruited 18 clusters, but the interest of the participants within the groups was much lower than the expected, resulting in an average recruitment of 3.8 participants per cluster. Because of the small size of the clusters and the variance in their size, in the following analyses we did not take into account the clusters but analyzed the population in two groups (tailored and control)."

7b) CONSORT: When applicable, explanation of any interim analyses and stopping guidelines

N/A

8a) CONSORT: Method used to generate the random allocation sequence

The randomization of clusters was based on a true random number online service, and participants were blinded to the group assignments.

8b) CONSORT: Type of randomisation; details of any restriction (such as blocking and block size)

"In practice, we recruited 18 clusters, but the interest of the participants within the groups was much lower than the expected, resulting in an average recruitment of 3.8 participants per cluster."

9) CONSORT: Mechanism used to implement the random allocation sequence (such as sequentially numbered containers), describing any steps taken to conceal the sequence until interventions were assigned

"The participants were instructed by the personnel of the rehabilitation center to use a specific number (PIN) that would automatically allocate them to their monthly cluster."

10) CONSORT: Who generated the random allocation sequence, who enrolled participants, and who assigned participants to interventions

"The participants were instructed by the personnel of the rehabilitation center to use a specific number (PIN) that would automatically allocate them to their monthly cluster."

11a) CONSORT: Blinding - If done, who was blinded after assignment to interventions (for example, participants, care providers, those assessing outcomes) and how

11a-i) Specify who was blinded, and who wasn't

"The participants were blinded to their study allocation. The investigators and outcome assessors were also blinded to the group assignments; however, for quality assurance related to technical issues, they had to uncover the assignments early during the statistical analysis process"

11a-ii) Discuss e.g., whether participants knew which intervention was the "intervention of interest" and which one was the "comparator"
"The participants were blinded to their study allocation. The investigators and outcome assessors were also blinded to the group assignments; however, for quality assurance related to technical issues, they had to uncover the assignments early during the statistical analysis process"
11b) CONSORT: If relevant, description of the similarity of interventions
N/A
12a) CONSORT: Statistical methods used to compare groups for primary and secondary outcomes
"we used the Kolmogorov-Smirnov Z with an exact calculation of the significance to compare the intervention with the control group. As an indicator of the effect size of the Kolmogorov-Smirnov Z comparisons, we calculated the strength of association, r. For the analysis of the categorical data, we used a chi-square test with an exact calculation of the significance and presented the effect of the size with the phi coefficient (φ). We used analysis of variance (ANOVA) for the scale variables at baseline that were found to be normally distributed since parametric tests have higher power and we did not want to miss statistically significant differences that would indicate that the two groups are not equal at baseline. For the effect size of the ANOVA comparisons, we used eta squared ($\eta 2$)."
12a-i) Imputation techniques to deal with attrition / missing values
"To maximize the use of our data, we included all the cases with valid data per time-point and per variable."
12b) CONSORT: Methods for additional analyses, such as subgroup analyses and adjusted analyses
N/A

RESULTS

13a) CONSORT: For each group, the numbers of participants who were randomly assigned, received intended treatment, and were analysed for the primary outcome
A flow diagram of the study is submitted.
13b) CONSORT: For each group, losses and exclusions after randomisation, together with reasons
A flow diagram of the study is submitted.
13b-i) Attrition diagram
Attrition diagram is included.
14a) CONSORT: Dates defining the periods of recruitment and follow-up
"The data were collected from January 2012 until October 2013"
14a-i) Indicate if critical "secular events" fell into the study period
N/A
14b) CONSORT: Why the trial ended or was stopped (early)
N/A
15) CONSORT: A table showing baseline demographic and clinical characteristics for each group
It is included.
15-i) Report demographics associated with digital divide issues
It is included in a table.
16a) CONSORT: For each group, number of participants (denominator) included in each analysis and whether the analysis was by original assigned groups
16-i) Report multiple "denominators" and provide definitions
It is included in tables.
16-ii) Primary analysis should be intent-to-treat
Primary analysis was not intention-to-treat. According to the latest CONSORT update this is not a requirement, and in our intervention, with high attrition, it would be unreliable.
17a) CONSORT: For each primary and secondary outcome, results for each group, and the estimated effect size and its precision (such as 95% confidence interval)
Included on tables.
17a-i) Presentation of process outcomes such as metrics of use and intensity of use
"The adherence rate 50 days after baseline was 48.3% for the tailored group and 68.4% for the control group (Figure 7). At 146 days after baseline, both groups had similar adherence rates of 41.0%, and from then on, the tailored group seemed to maintain a slightly higher level of adherence. At one year from baseline, the adherence rate was 25.6% for the tailored group and 24.0% for the controls. The mean adherence time for the tailored group was 176.3 (100.3–252.4) days and 177.9 (119.1-236.6) days for the control group; these findings were not significantly different (Breslow $\chi 2=0.725$, $p=0.395$). The mean adherence time for men was 207.9 (132.2–228.2) days and 92.5 (56.4–128.6) days for women; these values were significantly different (Log Rank $\chi 2=4.206$, $p=0.040$)."
17b) CONSORT: For binary outcomes, presentation of both absolute and relative effect sizes is recommended
Yes, in tables and in text.
18) CONSORT: Results of any other analyses performed, including subgroup analyses and adjusted analyses, distinguishing pre-specified from exploratory
N/A
18-i) Subgroup analysis of comparing only users

19) CONSORT: All important harms or unintended effects in each group
N/A
19-i) Include privacy breaches, technical problems
"Our intent was to measure the number of logins, time spent logged in, and what elements were used most for each participant. Due to a technical issue, the "time spent logged-in" data that we collected was not reliable. Instead, we used the time between the first and last login as the duration of the website use. We suspect there may have been issues with the number of logins per user as well, but in this case, the problem affected only a small portion of the users for a limited period of time."
19-ii) Include qualitative feedback from participants or observations from staff/researchers
N/A

DISCUSSION

20) CONSORT: Trial limitations, addressing sources of potential bias, imprecision, multiplicity of analyses
20-i) Typical limitations in ehealth trials
"Our sample was small, so we believe that our comparisons did not have enough power to confidently detect the effect of the intervention. Despite our efforts, the recruitment of participants was not at the desired levels, mainly because of the age of the participants of the cardiac rehabilitation program we were recruiting from."

21) CONSORT: Generalisability (external validity, applicability) of the trial findings
21-i) Generalizability to other populations
"Our approach, like that of many others [51–53], was not successful in addressing the needs of women, therefore our results cannot be generalized to both genders. "
"The inclusion criteria of our study were very broad, allowing for the recruitment of participants within a wide age range with a variety of comorbidities. This makes it more difficult to demonstrate the effect of the intervention since it is more difficult to affect the health behavior of patients with more complicated cases or older people and more difficult to isolate the effect of the intervention in a carefully selected population. However, this makes our study a real-world trial that will help us understand if and how the intervention is helping the population that needs it.2
21-ii) Discuss if there were elements in the RCT that would be different in a routine application setting
Real-world study, mostly can be used as it is.
22) CONSORT: Interpretation consistent with results, balancing benefits and harms, and considering other relevant evidence
22-i) Restate study questions and summarize the answers suggested by the data, starting with primary outcomes and process outcomes (use)
"Overall, the participants in our intervention moved forward through the stages of change following their rehabilitation stay; at discharge, about half of the participants were in the contemplation stage whereas three months after discharge, half of the participants were in the action stage. Despite the fact that half of the participants received a version of the intervention that was tailored to the stage of change, there were no differences between the groups with respect to their stage progressions. There was, however, a clinically meaningful as well as statistically significant difference between the groups in how well they were able to maintain their total physical activity. After discharge, the tailored group began increasing their physical activity after an initial drop, whereas the control group's physical activity decreased. This trend continued at three months after discharge; the physical activity of the tailored group continued to increase, whereas the physical activity of the control group continued to decline.
As the stage of change results suggest, this intervention might not have worked through the hypothesized mechanisms. The participants in the tailored group did not perceive their intervention as more personally relevant than the participants in the control group perceived theirs, and they did not consider the tailored messages received by email and SMS or the tailored questionnaires as particularly useful. In the beginning of the intervention, there was a higher drop-out rate in the tailored group than in the control group, although the average time until drop-out for the two groups was the same. Furthermore, the participants in the tailored group reported slightly lower self-efficacy than the control group and about the same level of perceived social support as the control group."
22-ii) Highlight unanswered new questions, suggest future research
"To increase the participation and adherence of women, we should have investigated more thoroughly any gender-specific barriers and needs."
"however, the trends from our findings indicated that tailored intervention holds promise for supporting the maintenance of long-term physical activity after cardiac rehabilitation. "

Other information

23) CONSORT: Registration number and name of trial registry
www.clinicaltrials.gov: NCT01223170.
24) CONSORT: Where the full trial protocol can be accessed, if available
http://www.biomedcentral.com/1471-2261/12/50 Included as reference.
25) CONSORT: Sources of funding and other support (such as supply of drugs), role of funders
"The project was fully funded by a PhD grant of the Northern Norway Regional Health Authority (Helse Nord RHF, ID 3342/HST986-10).2
X26-i) Comment on ethics committee approval
"The study protocol was approved by the Regional Ethics Committee for health region NORD (REK-NORD), and all the participants signed a consent form before being included in the study. "
x26-ii) Outline informed consent procedures
"Those who were interested met later to receive additional information, complete the consent form in paper and receive training in the use of the intervention."
X26-iii) Safety and security procedures
"Those who were interested met later to receive additional information, complete the consent form in paper and receive training in the use of the intervention. "
X27-i) State the relation of the study team towards the system being evaluated
"The authors have participated in the design of the interventions described in the manuscript."
